# Placental Site Trophoblastic Tumor: A Case Report and Review of the Literature

**DOI:** 10.3389/fsurg.2014.00031

**Published:** 2014-08-27

**Authors:** Jean Bouquet de la Jolinière, F. Khomsi, Anis Fadhlaoui, Nordine Ben Ali, Jean-Bernard Dubuisson, Anis Feki

**Affiliations:** ^1^Unit of Surgical Oncological Gynecology, Clinic of Gynecology and Obstetrics, Cantonal Hospital of Fribourg, Fribourg, Switzerland

**Keywords:** Immunochemistry, trophoblastic tumor, placental site, chemotherapy, pregnancy

## Abstract

Placental site trophoblastic tumor is rare. They represent a rare form of gestational trophoblastic disease. They occur mainly in women who have a history of miscarriage, termination of pregnancy, or even a normal or pathological ongoing pregnancy. The clinical course is unpredictable. This malignancy has different characteristics from other gestational trophoblastic tumors. Following a clinical case that we encountered and treated, we conducted a literary research and review, focusing primarily on prognostic factors and treatment.

## Introduction

Placental site trophoblastic tumor (PSTT) is a rare tumor, representing from 0.23 (1) to 3% (2) of gestational trophoblastic diseases (GTD). “Up to date” state that 300 cases have been reported in the literature (3). It mainly affects women of childbearing age, after pregnancy.

Placental site trophoblastic tumor differs from other GTD by a slow growth and a relative resistance to chemotherapy.

## Clinical Case

A 30-year-old patient, gravida 1 and para 0, gave birth naturally and without complications to a boy weighing 3290 g in May 2011 after a previous normal delivery in 2008 of a girl weighing 2970 g. Five months after her second delivery, the patient presented with bleeding (metrorrhagia). The pelvic echography showed no distinctive characteristics. A slightly high level of beta-HCG was observed (see Table 1). The presumed diagnosis was a trophoblastic retention. An operative hysteroscopy allowed the removal of 5 cc material. Its histology demonstrated the presence of several trophoblastic viable cells or nearly pyknotic trophoblastic looking cells without signs of malignancy. A weekly follow-up of beta-HCG was instituted, and the patient was treated with an oestroprogestative contraception.

**Table 1:** **Evolution of beta-HCG levels**.

Date	beta-HCG (UI/ml)
23.04.2012	103
02.05.2012	89
30.05.2012	104
09.07.2012	58
24.07.2012	45

Given the stagnation of beta-HCG during 2 weeks, a further operative hysteroscopy with 3 cc of product removal was carried out. After proofreading the blades and further immunohistochemical study, the diagnosis of PSTT was elected.

The extensive workup included a thoracic-abdominal-pelvic CT, which showed a large sized uterus displaying a heterogeneous rising with moderate bilateral ecstasy of the uterine veins. A cerebral MRI and a PET-CT did not detect the presence of metastasis. After discussion with the couple, a total hysterectomy with preservation of ovaries was decided. Intraoperative exploration is unremarkable except for bilateral uterine venous ecstasy.

**Figure 1:**
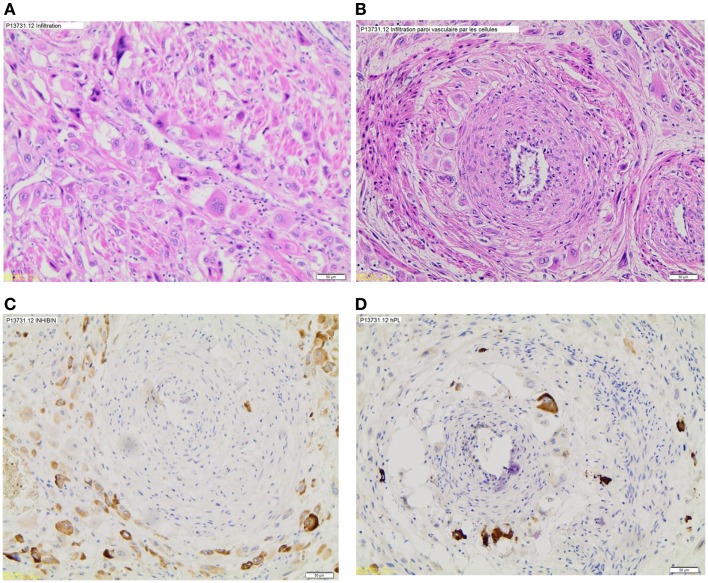
**Histologic sections**. **(A)** Myometrium infiltration by tumor cells, **(B)** vascular invasion, **(C)** inhibin marking, and **(D)** HPL marking.

Final histology confirmed a PSTT with a diameter of 23 mm, with infiltration of > 50% of the myometrium. The immunohistochemistry showed strongly positive tumorous staining for inhibin, partially positive staining for beta-HCG and for HPL (placental lactogenic hormone), and negative staining for p63. In addition, the Mib-1(Ki67) has reached 10–15% by locations (see Figure 1).

## Discussion

Placental site trophoblastic tumor is a subtype of GTH. These include partial and complete hydatidiform moles, invasive moles, choriocarcinoma, PSTT, and epithelioid trophoblastic tumors (TTE). The last three potential entities are grouped under the term gestational trophoblastic neoplasia, given their potential malignancy (4).

The cell proliferation in GTD comes from the placental trophoblast. It consists of syncytiotrophoblast, cytotrophoblast, and intermediate trophoblast. The syncytiotrophoblastic cells are polynuclear cells that form the external layer, mainly produce human chorionic gonadtropin (HCG) and invade the endometrial stroma. The cytotrophoblasts form the basal layer, whose cells can differentiate in syncytiotrophoblastic cells or intermediate trophoblastic cells. The intermediate trophoblastic cells leave the placenta to restructure the spiral arterioles in order to decrease the blood flow resistance toward the placenta (4, 5). PSTTs come from these spiral arterioles (6).

Histologically, PSTTs consist in a proliferation of intermediate trophoblastic cells without chorionic villi infiltrating muscle fibers (7). They are characterized with a vascular invasion, a necrosis, and hemorrhage at a lesser extent than choriocarcinoma, and a bigger tendency to disseminate through the lymphatic track (3, 4).

The immunohistochemical analysis shows a strongly positive staining for HPL; a generally weak and focal positive staining for HCG (3, 7, 8); a diffuse positive staining for cytokeratin (7), a strong positive staining for epidermal growth factor receptor (EGFR) and vascular endothelial growth factor (VEGF); and a negative staining for human epidermal receptor2/neu (HER2/neu) and cluster of differentiation117 (CD117) (3). The pregnancy-associated major basic protein (pMBP), a marker of the intermediate trophoblast, turns out useful in differentiating PSTT from other forms of TTE (9).

As for the tumor’s pathogenesis, this remains to be clarified. Hui and colleagues found an absence of Y-chromosome in 20 cases of PSTT with a haploid pair of X chromosomes. They suggest the paternal X chromosome has a functional role in this tumor, with a more important expression of the genes responsible for growth in the paternal genome (6). Other authors report a predominance of female fetuses (11 female fetuses for 2 male ones) among the pregnancies carried to term that were the source of a PSTT (1).

The first case of PSTT was described in 1976 by Kurman and colleagues on a series of 12 cases, referred to as “trophoblastic pseudo tumor,” for a supposed benign pathology. Since then, just fewer than 300 cases have been described in the literature, with an assured potential for malignancy. The terminology was therefore adapted to the current PSTT denomination in 1981, through the works of Scully (10).

Placental site trophoblastic tumor can arise during or after a normal pregnancy, a miscarriage, an abortion, or a molar pregnancy, and can occur during post-menopause, years after the last pregnancy ((1, 3, 7), and (11)). A case describes multiple metastases in a 4-month-old male infant who consequently died (12).

The primary tumor site is nearly always located in the corpus or the fundus of the uterus, but two cases of cervical location have been described (13).

The patients’ age is between 20 (14) and 63 years old (3), 32 years old on average (7). The gap since the last pregnancy is on average 34 months (median of 18 months) after the last-known pregnancy.

In the literature, the mean gestity is 2, 2 (14).

Symptomatology is variable. It can be amenorrhea (1, 14), bleeding ((14, 15), (1)), uterine rupture (1), abdominal pain (1), post-menopausal bleeding (1), virilization (16), or symptoms due to metastases (1, 17). Cases of nephritic syndrome on membranous glomerulonephritis have been described, regressing after treating the tumor (18, 19). Some cases were asymptomatic and only the persistent increase in beta-HCG levels led to the diagnosis (14).

The stage of the illness is determined by the gestational TTE FIGO stage (see Tables 2 and 3).

**Table 2:** **Anatomical classification FIGO 2000**.

I	Limited to the uterus
II	Extended outside the uterus but limited to genital structure
III	Extended to lungs with or without known genital tract reached
IV	Any other site of metastatis

**Table 3:** **Stage of the disease at diagnosis**.

Studies	No. of cases	Stage I (%)	Stage II (%)	Stage III (%)	Stage IV (%)	Non-set (%)
Chen et al. (15)	17	88.2	0	11.8	0	
Feltmate et al. (14)	13	69.2	30.8	0	0	
Baergen et al. (7)	55	84	2	5	9	
Lan et al. (3)	5	40	40	20	0	
Hassadia et al. (1)	17	47.1	5.9	29.4	17.6	
Chang et al. (20)	88	65.9	4.5	11.4	12.5	5.7
Schmid et al. (8)	62	55	8	26	11	
Hoekstra et al. (28)	7	57	0	0	43	
Papadopoulos et al. (23)	34	44	24	29		3
Shen et al. (32)	6	100				
Zhao et al. (37)	11					

The majority of diagnosed cases are at stage I in the classification of GTD according to FIGO (limited to the uterus), 14 (7) to 31% (20) of the cases initially with metastases manifested.

The metastases sites are (from most frequent to rare): lungs (7), liver (1, 7), vagina (1, 7), gastrointestinal tract (7), brain (1, 7), lymph nodes (3, 7), bladder (7), ovary (7, 21), omentum (7), thoracic diaphragm (7), pancreas (1, 7), spleen (7), kidney (1), bone marrow (7), and scalp (22).

The tendency to metastasize through lymphatic ways seems significant, as 5.9% of cases have lymphatic metastases during diagnosis or at the time of recurrence. The lymphatic metastases sites are (from most frequent to least frequent): para-aortic (50%), pelvic (35.7%), non-specified retroperitoneal (14.3%), inguinal (7.1%), renal hilum (7.1%), mesenteric (7.1%), mediastinal (7.1%), hilar (7.1%), and supraclavicular (7.1%) (3). Lan and colleagues suggest to conduct a retroperitoneal lymphadenectomy on stage 1 patients who have risk factors such as myometrium invasion > 50% as well as all stages II patients. For other stages, the lymphadenectomy depends on the location and on the resect ability of the other metastases (3).

When looking at the biology, the beta-HCG levels are moderately high, unlike the levels found in the hydatiform moles. In 79% of cases, the level is lower than 1000 IU/l and in 58% of cases, it is lower than 500 IU/l (23).

Through a routine trans-vaginal ultrasound, one can see the tumor or not, it can have a solid or cystic aspect (24, 25). The invasion and destruction of the myometrium vascularization can cause the formation of blood deficiencies and of artery-venous shunt with hemodynamic modifications (26). Some authors have noticed that the tumor vessels have low resistance with high diastolic velocity (25, 26). Bettencourt and colleagues suggest a Doppler monitoring to monitor the response to chemotherapy as well as a monitoring of the beta-HCG level (26).

On the MRI, the specifications of PSTT are non-specific (27). In the case of tumors that are invisible on the ultrasound, MRI is particularly interesting to locate the tumor and determine the loco-regional extension, thus allowing guidance of treatment (27).

Placental site trophoblastic tumor behavior is characterized by a slower growth, when compared to other TTE, as well as a tendency to diffuse through lymphatic ways. Therefore, they are relatively resistant to chemotherapies (3).

The preferred treatment for PSTT is essentially surgical and is based on hysterectomy (1, 14, 15, 28) with lymphatic sampling (8, 29). Conducting a lymphadenectomy at stage 1 seems particularly appropriate in case of risk factors such as a myometrium invasion superior to 50% (3). Please note that the lymphatic metastases are not listed in the FIGO classification system. The hysterectomy can be inter-adnexal on pre-menopausal patients without a high ovarian carcinoma family risk (8, 24).

Several cases of located tumor resection, with the aim to preserve fertility, have been described (30–32). In many cases, an additional hysterectomy was consequently required (inadequate or invaded resection margins, beta-HCG post-operative increase with diffuse myometrium infiltration) (31). In the series described by Saso and colleagues, a case of partial resection through a modified Strasmann procedure allowed to preserve the fertility of a patient. A patient described by Tsuji and colleagues was also treated in such a way as to conserve the uterus with neo-adjuvant chemotherapy by EMA/CO and then tumor resection by laparotomy (30). Shen and colleagues describe six patients successfully treated by a combination of surgery and chemotherapy with uterine conservation. A pregnancy carried to term was reported among one of these patients (32).

In any case, the success level of a conservative treatment is low and this option should be proposed only after an extensive discussion and detailed patient information. It should be reserved for young patients not finding any poor prognosis factors and whose tumor is focal (32).

As far as adjuvant therapies are concerned, Schmid and colleagues have not demonstrated the benefit of chemotherapy for stage I patients (8). On the other hand, a combined surgery and chemotherapy treatment is recommended from stage II onward (8, 28). For stage I cases, with poor prognosis factors, such as a long-time interval since the last pregnancy (28), a high mitotic index [Hoekstra et al. – (28)], a vascular invasion, a deep myometrium invasion, an invasion of the serous membrane, or for cases with persistence of high post-operative beta-HCG levels, chemotherapy could also be recommended (8, 14, 29). In a series of cases, Feltmate and colleagues found that the prognosis was improved when starting chemotherapy during the week following the operation (14).

In comparison with other gestational TTE, the response to chemotherapy is lower in PSTT, with 61% resistance or incomplete response (8).

Comparative data from different chemotherapy schemes are missing. The ones that are generally administrated are: EMA/CO (7) or EMA/EP (8, 14, 24, 29), without a difference being revealed between the EMA/EP, EMA/CO, or EMA schemes, or between the diets with or without platines (8) (see Table 4).

**Table 4:** **Protocol of chemotherapy**.

EMA/EP	EMA	0.5 mg dactinomycin iv J 1 et 2
		100 mg/m^2^ etoposide iv J 1 et 2
		300 mg/m^2^ methotrexate iv J1
		15 mg folic ac po 2x/j J 2 et 3
	EP	150 mg/m^2^ etoposide iv J8
		75 mg/m^2^ cisplatin iv J8
MAE		300 mg/m^2^ methotrexate iv J1
		15 mg folic ac po 4x/j J 2 et 3
		0.5mg dactinomycin iv J 8 à 10
		100 mg/m^2^ etoposide iv J 8 à 10
BEP		bleomycin 30 mg iv J1, 8, 15
		etoposide 100mg/m^2^ J1 à 5
		cisplatin 20 mg/m^2^ J1 à 5
VIP		etoposide 75 mg/m^2^ J1 à 5
		ifosfamide 1.2 g/m^2^ J1 à 5
		cisplatin 20 mg/m^2^ J1 à 5

For second-line therapies, there is no consensus, the EMA/EP scheme is proposed in the case of recurrences after EMA/CO (7), or BEP (7, 33) or VIP (7) schemes.

The resection of residual lumps after chemotherapy is recommended, given the lower sensitivity of tumor cells after chemotherapy, which is confirmed by presence of viable tumor cells in the specimen resected after primary chemotherapy (8, 34, 35).

According to Feltmate and colleagues, radiotherapy can be effective for loco-regional control and remains rarely recommended (14).

The factors that are most strongly correlated with poor prognosis, which were found in almost all series, are a high stage according to FIGO (1, 7, 8, 20, 28) (Table 5). The value of the FIGO risk scores is controversial (Table 6). According to Hassadia and colleagues, it should not be used (1, 36), but Schmid and colleagues’s study shows a correlation with survival (8).

**Table 5:** **Prognostic scores (FIGO 2000)**.

	0	1	2	4
Age	<40	≥40	–	–
Previous pregnancy	Mole	Abortion	Pregnancy on term	–
Delay between the end of pregnancy and the beginning of chemotherapy	<4 mois	4–7	7–13	>13
Plasmatic HCG before the treatment (IU/l)	<10^3^	10^3^–10^4^	10^4^–10^5^	>10^5^
Larger tumor	–	3–5	>5	–
Metastatic sites	Lang (standard X Ray)	Kidney, spleen	Bowel, ileon	Brain, liver
Number of metastatis	0	1-4	5-8	>8
Failure of prior chemo therapy	–	–	Mono-chemotherapy	Poly-chemotherapy

**Table 6:** **Analysis of literature**.

Chen et al. (15)	17 Cases	Hysterectomy with or without EMA-CO chemotherapy is a beneficial treatment modality.
Feltmate et al. (14)	13 Cases	High mitotic index appears to be an adverse prognostic indicator for recurrence. Hysterectomy remains the mainstay of treatment. Chemotherapy is indicated for patients with metastases and may be indicated when the mitotic index is > 5 Mitoses/10 HPF. Radiation treatment may play a role in recurrent disease but must be evaluated on a case-by-case basis.
Baergen et al. (7)	55 Cases	Significant factors associated with adverse survival in the present series were age over 35 years (*P* = 0.025), interval since the last pregnancy of over 2 years (*P* = 0.014), deep myometrial invasion (*P* = 0.006), stage III or IV (*P* < 0.0005), maximum high HCG level > 1000 mIU/ml (*P* = 0.034), extensive coagulative necrosis (*P* = 0.024), high mitotic rate (*P* = 0.005), and the presence of cells with clear cytoplasm (*P* < 0.0005).
Lan et al. (3)	5 Cases	Lymph node metastasis is one way of spread in PSTT. Retroperitoneal node, especially para-aortic node is the most common site of lymphatic spread. EGFR and VEGF may be commonly expressed in PSTT tumors.
Hassadia et al. (1)	17 Cases	Hysterectomy is the primary mode of treatment in the majority of cases. However, chemotherapy can still play a major role when curative surgery is not feasible
Chang et al. (20)	88 Cases	FIGO stage is the most important prognostic factor, and complete removal of all lesions provided good outcomes in PSTT patients. For those with unresectable tumors, combination chemotherapy showed a high response rate, but only a few achieved a complete response.
Schmid et al. (8)	62 Cases	Stage-adapted management with surgery for stage I disease, and combined surgery and chemotherapy for stage II, III, and IV disease could improve the effectiveness of treatment for placental-site trophoblastic tumors. Use of 48 months since antecedent pregnancy as a prognostic indicator of survival could help select patients for risk-adapted treatment.
Hoekstra et al. (28)	7 Cases	Advanced FIGO stage, long interval from last known pregnancy to diagnosis, and high mitotic count were adverse prognostic indicators for survival in PSTT. All patients with PSTT should undergo initial hysterectomy with other surgical procedures, as indicated. Chemotherapy, usually EMA/EP, should be used in patients with advanced PSTT and may be considered in patients with FIGO stage I disease with length of time from antecedent pregnancy > 2 years or high mitotic.
Papadopoulos et al. (23)	34 Cases	Risk factors for death include lung metastatic involvement (50%) and an antecedent pregnancy interval of 4 years or more (100%). In contrast, those with no extra pelvic disease or a pregnancy interval of less than 4 years had 100% survival. In two-thirds of patients with disease limited to the uterus, surgery alone was curative.
Shen et al. (32)	6 Cases	Fertility-conserving therapy for young women with PSTT would be practicable if the patient is younger than 35 years, strongly desires to preserve fertility and responds well to chemotherapy and conservative surgery, the pathological results of which do not show poor prognostic factors and the gross pathologic type does not present markedly enlarged uterus, diffuse infiltrative, and diffuse multifocal disease within the uterus.
Zhao et al. (37)	11 Cases	Pathologic diagnosis of PSTT was the gold standard. Multidrug chemotherapy combined with hysterectomy was effective in metastasis cases.

Other poor prognosis factors are an interval of more than 24 months since the last pregnancy (7, 8, 23, 28), a level of mitosis higher than 6 in 10 bigs fields (7, 8, 14), an age older than 34 years (1, 7, 8), a term birth for the last pregnancy (7), a myometrium invasion of more than 50% (7), an extensive coagulation necrosis (7), and cells with clear cytoplasm (7).

A beta-HCG level higher than 1000 IU/l (7, 8) is also a poor prognosis factor. On the other hand, this rate is not correlated to the tumor mass (1).

## Conclusion

Placental site trophoblastic tumors are exceptional tumors, encountering difficult clinical and histological diagnosis. Immunohistochemistry plays an important role, and our case underlines the importance of reading through the blades several times.

Clinically speaking, their emergence is seldom or not at all predictable because their generally appear after a normal pregnancy.

The usual symptoms are non-specific, such as metrorrhagia or amenorrhea, sometimes years after the last pregnancy. From a biological point of view, the dosage and follow up of beta-HCG levels are interesting for the diagnosis, but one must keep in mind that the levels are not as high as in other GTH.

The only FIGO classification criticism for GTHs is that the lymphatic spread is not taken into account.

In the case of PSTT, the most commonly recognized risk factors are: stage, mitosis rate, elapsed time since last pregnancy at the time of diagnosis, age of the patient, and the degree of myometrium invasion.

With regard to treatment, the leading role of surgery must be underlined, generally through hysterectomy. The possibility of a treatment conservative of fertility can be discussed with patients who wish to become pregnant again, have high motivation, good prognosis factors, and a thorough discussion.

A lymph node sampling is generally recommended. Chemotherapy is usually not recommended at stage I, but can play a role in cases of poor prognosis. Currently, there is no consensus on the best chemotherapeutic treatment.

## Conflict of Interest Statement

The authors declare that the research was conducted in the absence of any commercial or financial relationships that could be construed as a potential conflict of interest.
